# Resistance integrons; A Mini review

**DOI:** 10.22088/cjim.10.4.370

**Published:** 2019

**Authors:** Fariba Akrami, Mahdi Rajabnia, Abazar Pournajaf

**Affiliations:** 1 Department of Microbiology, Faculty of Medicine, Babol University of Medical Sciences, Babol, Iran; 2 Infectious Diseases and Tropical Medicine Research Center, Health Research Institute, Babol University of Medical Sciences, Babol, Iran

**Keywords:** Integron, gene cassettes, antibiotic resistance

## Abstract

Integrons are a segment of dsDNA that play a major role in bacterial adaptation and evolution. These genetic determinants are known by the presence of three necessary apparatuses: an integrase (*intI* gene), *Pc* (a promoter) and *attI* (a recombination site). These elements are able to acquire gene cassettes, which can carry antibiotic resistance factors, by site-specific recombination mechanism. The most common types of resistance integrons are class I (Tn402 derivatives), followed by class II and III. In recent years, the role of integrons as an important factor in the transmission and spread of resistance factors has been considered. Therefore, the ongoing threats posed by integrons require an understanding of their origins and evolutionary history. This review examines the functions and activities of integrons. It shows how antibiotics use selected particular integrons from the environmental pool, so that integrons carrying resistance genes are now present in the majority of Gram-negative pathogens.

Horizontal Gene Transfer (HGT) in the bacterial species means the movement of genetic material between bacteria from a similar genus (1). The HGT plays an important role in evolution, diversity, recombination and multi-drug resistant strains (2, 3). Antibiotic-resistant determinants in resistance bacteria are usually carried on mobile genetic elements (MGEs), such as the plasmids, transposons (TEs), integron (Int), and multidrug resistance genomic islands. Integrons are conserved dsDNA sequences (3′-CS and 5′-CS) of DNA that are able to obtain gene cassettes, which can carry drug resistance genes, by site-specific recombination. (4-8). In this review article we described an integron structure, types and their distribution in antibiotic resistance bacteria.

**Integrons and gene cassettes:** These elements are immobilized but located on the transferrable plasmids which can disseminate in the intra- and inter-bacterial species (figure 1).

These elements have three main components: (1) IntI which is encodes integrase gene and it is the Part of tyrosine kinases family. The coding protein derived from this gene plays an important role in the recombination of genetic cassettes. (2) aatI is recognized by the integrase, so is a receptor site for gene cassettes integration by site-specific recombination (10-13). (3) Pc is a promotor which necessary for the transcription and expression of genetic cassettes in integron (14 - 16). The circular gene cassettes are DNA segments which can integrate between attI and attC by integrase (figure 2). This process can also be reversible, leading to gene cassette deletion (17-19).

**Figure 1:**
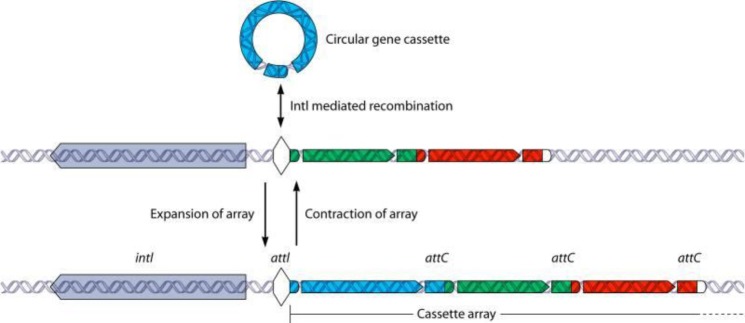
Transfer of antibiotic resistance elements through integrons: the figure gives a schematic representation of transmission of integrons. Transposons (Tn) containing integrons can transfer into a microbial strains from natural sources. The int1 and the att1 are corresponded for attachment and integration of the gene cassettes. Resistance to sulfonamides and quaternary ammonium compounds were encoded by the sul1and qacEΔ1 genes, respectively. The grey zones showed the gene cassettes with different functions. The Pint and Pc are integrase (int1) and gene cassettes promoters, respectively (9)

**Figure 2:**
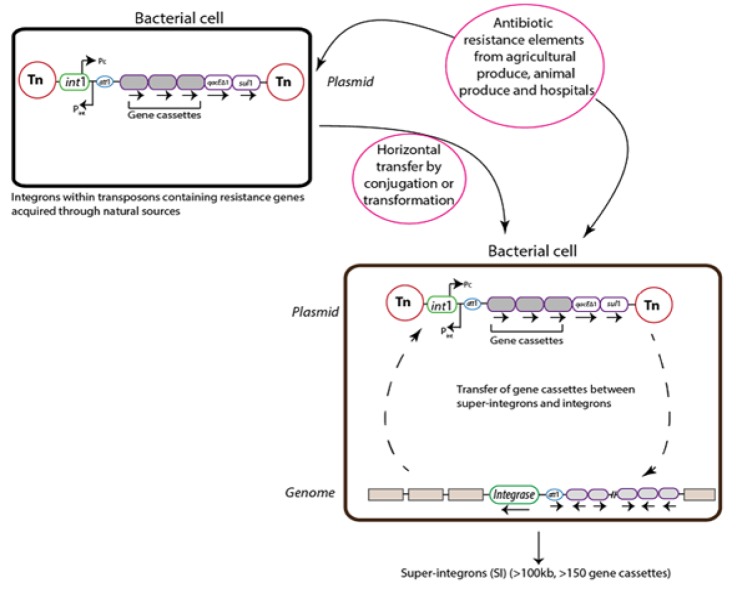
Acquisition of gene cassettes. Integrons obtain a new gene cassette by the site-specific recombination between attC in the circular gene cassette and attI site in integron. These insert cassettes are at the position proximal to int gene and its embedded promoter. Cassette arrays can enlarge by repeated cassette acquisition, but cassettes can also be excised as closed circles by attC × attI or attC × attC recombination (10)

**Integron types:**

Two main groups of integrons are identified including: mobile integrons (MIs) and chromosomal integrons (CIs). CIs are found in marine bacterial chromosome, such as Vibrio species. The other name for this class is Super-integrons (SIs), because they can carry more than 200 cassettes and usually encode proteins with unknown function (20). On the other hand, CIs can carry a numerous number of gene cassettes, which are commonly not involved in drug resistance. MIs are located on the mobile genetic elements (MGEs) such as plasmids and transposons, and carry only a few cassettes. MIs can carry antibiotic resistance cassettes, because of this function; they called resistance integrons (RIs) (21). 

**Resistance integrons**

These integrons are divided into five groups based on the similarity of amino acid sequences. These elements are most commonly found in gram-negative bacteria (22-26). Class I integrons differs widely between gram-negative bacteria and are transmitted through Tn402. 

The integron–integrase gene (intI1) is identical in all class I integron, and the left- hand end of all class I of integrons contains a conserved noncoding sequence that terminates in a 25-bp sequence (IRi), which is an inverted repeat of another sequence (IRt) located at the right-hand end of most extant class 1 integrons (27). The common ancestor of all clinical class I of integrons was likely same to a Tn402-like transposon, consisting of IRi, intI1, attI1, gene cassette(s) and their associated recombination sites (attC, or59-base elements), a qacE cassette (quaternary ammonium compound E; which encodes resistance to biocides), a complete transposition module and IRt, respectively (28). The class II of integrons has a *IntII* gene which is terminated by stop codon. This class is transmitted by Tn7 and its derivatives (29). The integrons class III is less important but found in clinical isolates and transmitted by Tn402 (30, 31). The integrons class IV and V are found in *Vibrio* and PRSV1 plasmid.

**Evolutionary History**

SIs are present as the pioneers of the old incubators (30). In contrast to other integrons, SIs are always related to chromosomes. They are large in size and carry more than twenty gene cassettes. For the first time, they were found in the small chromosome of* Vibrio cholerae *but today they are found in many bacterial species (32-34). Gillings et al, detected *intI1*gene from the environmental samples including, sediments, soils, and biofilms (27).

These MGEs are classified into broad groups based on the phylogeny of the *int* gene: (1) proteobacteria that found in water and soil, which mostly consists of integrons class I and III (2) γ-Proteobacteria, found in marine environments are includs the class II integrons (3) Integrons that integrase gene have revers originate with above categories are found in *Spirochetes* and *Acinetobacter*. The categorization of integrons based on the type of environment, contrary to their identification by the host, indicates that the transfer of integrons between species occurs in similar environments . These findings are based on phylogenic tree of 16sRNA and Int1 (14,30,35). The miniature inverted-repeat transposable elements (MITEs) are a group of non-autonomous Class II transposable elements found in *Acinetobacter* and *Enterobacter *that rearrangement or displacement of these regions can cause integron transfer in the chromosome**.** (36,37).

**-Integrons and antibiotic resistance**

Integrons acts as a pool of gene cassettes. These cassettes play a role in antibiotic resistance. In general, about 130 different genetic cassettes have been identified which are different in the codon patterns and attC site. These cassettes are usually small in the mobile integrons. The longest known row of gene cassettes is 8 gene cassettes. Perhaps one of the reasons is that the cassettes are controlled by a promoter (38, 40).

**Class I integron**

The left conserved sequence is 107- bp that is located under Int1 stop codon and the right conserved sequence is 43-bp is located under aatC site. Class I integrons harbor various antimicrobial resistance gene cassettes encoding dfr (dihydroflavonol-4-reductase), broad-spectrum β-lactamase, qacEΔ1 (quaternary ammonium compound disinfectant), sul1 (sulfonamide), and aminoglycoside-modifying enzymes (AMEs). Recently, its hybrid types consist of class I integron and cassettes, which still carry inverted repeat sequences (41-43). Class I integrons can capture and distribute gene cassettes among other integron classes. This broadcast is happened via the natural conjugation or transformation (44, 45). This class has been observed in gram-negative organisms such as Acinetobacter, Aeromonas, Alcaligenes, Burkholderia, Campylobacter, Citrobacter, Pseudomonas, Klebsiella, Salmonella (46-52). The studies are indicating a strong relation between cassettes and expression of the integrase (53-62).

**Class II integron**

Similar to the class I integron, class II integron is also found on Tn7 transposon family. Its 3′ conserved section (3′-CS) contains 5 *tns* genes, which act in transposon movement. Compared to Class I integrons, the *int* gene in the class II is less active and, consequently, class II is more limited in gene cassette acquisition. In addition, these integrons can carry unusual cassettes that encode the lipoprotein signal peptidase (63). The sequence of amino acid in class II is less than 50% identical to class I integron and this is due to the displacement of internal stop codon at position 179 in triplet coding for glutamic acid. This integron has *Dfr1*, *sul1*, *aadA1* (aminoglycoside adenylyltransferase) gene cassettes. This class is most commonly found in *Escherichia coli*, *Acinetobacter* *baumannii*, *Salmonella* *enterica*, and *Burkholderia* (64- 67).

**Class III integron**

It was first identified in 1993 by Arakawa et al, in Japan from *Serratia marcescens*. This class is transferred by Tn402, but it is not active as well as other classes. The 3' end of class III integrons are similar to the class I and contain the qacEΔ1, sul1 and orf genes, with the only difference being the lack of transposition genes in the class III. There are several cassettes in this class that include: bla-*IMP-1* (encodes for Metallo-β-lactamase enzymes), *aacA4* (tobramycin resistance gene). However, a class III integron had been recently identified containing blaGES-1 (an extended‐spectrum β‐lactamase (ESBL) encoded gene) within the IncQ plasmid from *E. coli*. To date, this class has been identified within a few microorganisms including *Escherichia coli*, *Acinetobacter* spp., *Salmonella* spp., *Citrobacter freundii*, *Alcaligenes*, *Klebsiella pneumoniae*, *Pseudomonas aeruginosa*, *Pseudomonas putida*, and *Serratia marcescens* (68, 69).

**Class IV integron**

It was identified in 1998 by Mazel et al. On the small chromosome of *Vibrio cholerae* and was named SIs. The size of this class is large (~126 kb) and contains at least 178 gene cassettes. This class have been found in *Vibrionaceae*, *Shewanella*, *Xanthomonas* and other marine proteobacteria group. These integrons are also found in Geobacter sulfurreducens, *Pseudomonas*, *Nitrosomonas*, *Listonella* and *Treponema* *denticol*. To date, class IV integrons have been found to carry gene cassettes imparting resistance to the fosfomycin and chloramphenicol (17, 23, 70-75).

It can be concluded that the gene cassettes in integrons are one of the compatible components of bacteria with environment. The ability of integrons to acquire new cassettes and their ability to recombined cassette rows emphasizes the adaptation of their diversity in bacteria. Integrons have the ability to carry cassettes with a variety of functions and are also compatible with the acquisition and expression of resistance components. In addition, most studies about integrons have been limited toclass 1 integron in gram-negative bacteria, but this class has also been observed in gram-positive bacteria. There are a lot of questions about the organisms carrying these integrons, gene cassettes, and the outbreak of them in particular species of bacteria.

## Conflict of Interest:

 The authors declare that there is no conflict of interest.

## Declarations:

**Ethical Consideration**

Ethical issues (Including plagiarism, informed consent, misconduct, data fabrication and/or falsification, double publication and/or submission, redundancy, etc.) have been completely observed by the author.
